# A multi-scale numerical approach to study monoclonal antibodies in solution

**DOI:** 10.1063/5.0186642

**Published:** 2024-02-26

**Authors:** Marco Polimeni, Emanuela Zaccarelli, Alessandro Gulotta, Mikael Lund, Anna Stradner, Peter Schurtenberger

**Affiliations:** 1 Division of Physical Chemistry, Lund University, Lund, Sweden; 2 Institute for Complex Systems, National Research Council (ISC-CNR), Piazzale Aldo Moro 5, 00185 Rome, Italy; 3 Division of Computational Chemistry, Lund University, Lund, Sweden

## Abstract

Developing efficient and robust computational models is essential to improve our understanding of protein solution behavior. This becomes particularly important to tackle the high-concentration regime. In this context, the main challenge is to put forward coarse-grained descriptions able to reduce the level of detail, while retaining key features and relevant information. In this work, we develop an efficient strategy that can be used to investigate and gain insight into monoclonal antibody solutions under different conditions. We use a multi-scale numerical approach, which connects information obtained at all-atom and amino-acid levels to bead models. The latter has the advantage of reproducing the properties of interest while being computationally much faster. Indeed, these models allow us to perform many-protein simulations with a large number of molecules. We can, thus, explore conditions not easily accessible with more detailed descriptions, perform effective comparisons with experimental data up to very high protein concentrations, and efficiently investigate protein–protein interactions and their role in phase behavior and protein self-assembly. Here, a particular emphasis is given to the effects of charges at different ionic strengths.

## I. INTRODUCTION

Therapeutic proteins, such as monoclonal antibodies (mAbs), represent an important tool for the treatment of numerous diseases, such as cancer,^1^ autoimmune disorders,^2^ and viral infections.^3^ An example is given by the anti-SARS-CoV-2 mAbs, recently authorized by the Food and Drug Administration (FDA), to be used for the treatment of COVID-19 symptoms, but more than 100 mAbs have already been approved^4^ in the last 35 years and many more are under investigation.^3^ However, antibody-drug development faces several challenges,^5^ such as protein aggregation, high viscosity, and phase separation. Protein aggregation, especially in the form of insoluble aggregates or high-molecular-weight species, can lead to reduced efficacy and potential immunogenicity.^6^ High viscosity and phase separation can pose challenges during manufacturing and storage processes.^7^ Therefore, understanding and controlling the mechanisms driving these phenomena is of fundamental importance. Several experimental techniques, such as static light scattering (SLS), dynamic light scattering (DLS), and small-angle x-ray scattering (SAXS), are commonly used to study these systems. SLS provides information about the size and molecular weight of aggregates or oligomers present in the solution. Furthermore, by analyzing the scattering pattern at different protein concentrations, SLS gives insight into protein–protein interactions and solution stability in terms of the osmotic compressibility *κ_T_* and the second osmotic virial coefficient, *b*_22_. DLS measures the intensity fluctuations of the scattered light caused by the Brownian motion of particles in a solution and is commonly used to determine the collective diffusion coefficient, the hydrodynamic radius, and the size distribution of monomers and aggregates. SAXS involves exposing a sample to x rays and analyzing the scattering pattern produced by the interaction of x rays with the sample. Therefore, SAXS provides structural information at much smaller length scales than SLS and gives valuable insights into the overall shape, size, conformational changes, and intermolecular interactions of proteins in solution. The combination of these approaches is usually adopted by taking a systematic variation of the formulation components, such as buffer conditions, temperature, pH, ionic strength, excipient concentrations, and surfactants, to identify the optimal conditions for mAb stability and solubility. Ideally, by monitoring changes in the aggregation state and size distribution of the aggregates, one can aim to optimize formulation conditions.

Although laboratory experiments are essential to investigate protein solutions, computational modeling is an invaluable tool to make progress in a multi-scale, challenging problem such as the study of antibody behavior in concentrated solutions. The combined use of computer simulations at different length scales can, thus, deliver important predictions on the best solution conditions. In particular, atomistic models combined with molecular dynamics (MD) and/or Monte Carlo (MC) simulations provide details on protein conformational changes and can be used to access time scales and length scales not directly observable through experiments. Furthermore, more coarse-grained models can be used to speed up the simulations and study the system at extended length and time scales and higher protein concentrations. Overall, computational modeling allows us to have control over properties, such as temperature, pressure, pH, and protein concentration. This level of control enables the investigation of the effects of specific conditions on protein behavior and helps to elucidate the underlying mechanisms and driving forces governing the solution properties. This strategy can be used to make predictions on structural and dynamic quantities inaccessible through experiments alone. Furthermore, computational modeling can be more cost-effective and time-efficient than certain experimental techniques. Performing experiments, especially those requiring specialized equipment, difficult sample preparation procedures, and large sample volumes, can be expensive and time-consuming. Furthermore, in the early stages of biologics development, the available quantities of material are usually limited, which makes systematic studies of the solution properties at high concentrations difficult or impossible. Computational modeling, thus, allows for virtual experiments and exploration of a wide range of conditions without physical materials and laboratory resources.

In a recent work,^8^ we investigated the solution properties of an IGg1 mAb, whose behavior is largely controlled by repulsive electrostatic interactions, by means of several experimental techniques (SLS, DLS, SAXS, and microrheology) and colloidal theory. For this system, we have shown that, even though the mAb charge distribution is heterogeneous, the dominant electrostatics is controlled by the mAb high net positive charge at low protein concentrations and low ionic strength. This suggested the use of a simple colloidal model for this mAb, where the protein is represented by a hard sphere with a homogeneous surface charge density resulting in an effective charge *Z_eff_* and an additional weak short-range attraction. The model was, indeed, capable of reproducing experimental properties, such as the apparent molecular weight or the collective diffusion coefficient over a large range of mAb concentrations and at different ionic strengths. However, this model failed to describe the more detailed structural properties of the mAb solution given by the static structure factor *S*(*q*) from SAXS for mAb concentrations higher than 50 mg/mL, because of the non-realistic charge treatment and the missing shape anisotropy of the underlying colloid model. We indeed showed in Ref. 8 that, to this end, the mAb shape anisotropy is needed to explore a wider range of solution conditions as previously done in several works.^9–12^

**FIG. 1.**
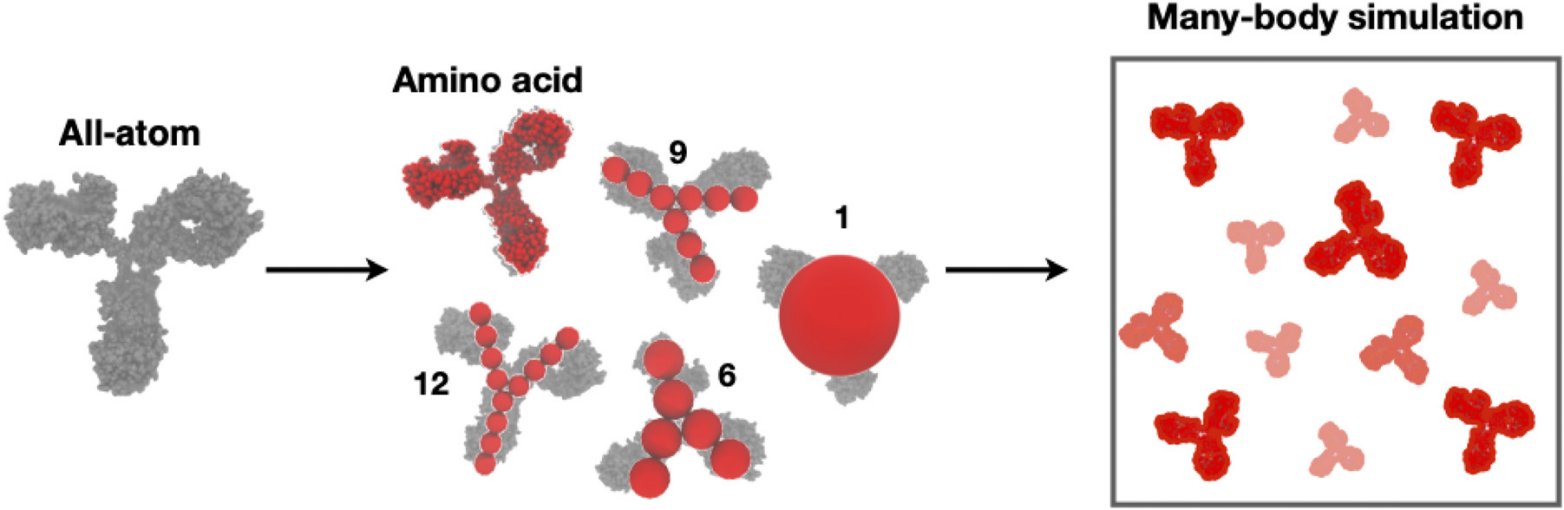
The experimental mAbs structure at atomic resolution (leftmost, gray) is first relaxed using MD simulations, where-after it is coarse-grained at five different levels shown in the middle, in red. This includes models with 1, 6, 9, or 12 interaction sites, as well as a model where each amino acid is treated as a (charged) sphere. The coarse-grained models are subsequently used in many-body MC simulations to explore the effect of varying mAb concentration and salt concentrations (rightmost).

Inspired by these considerations, in the present paper, we aim to overcome the limitations of using too simplified models while avoiding the computational costs of near atomistic simulations and develop the basis for a general protocol leading to efficient models able to describe solutions at high protein concentrations and high ionic strength. To do so, we go one step forward with respect to previous studies by working across different length scales. We, thus, use a multi-scale approach, described in detail below, which involves all-atom MD simulations, coarse-grained MC simulations, and mesoscale models, as shown in Fig. 1. By varying the mAb concentration, pH, and added salt, we explore several solution conditions and, for each of them, we can quantitatively reproduce the experimentally measured effective structure factor, $S_{\text{eff}}(q)$, obtained by SAXS. Such a comparison is highly non-trivial because it provides microscopic information about the spatial arrangement, conformational changes, and specificity of the interacting proteins, contributing to a better understanding of the underlying molecular interaction mechanisms. Analyzing these results, we rationalize the mAb behavior as a function of the different solution conditions. The starting point for our simulations is the all-atom conformation obtained with homology modeling using a commercial software package [molecular operating environment (MOE),^13^ see Sec. IV]. From this structure, we first build a coarse-grained version of the mAb at an amino-acid level and then perform constant-pH MC simulations to estimate the amino-acid charge distribution. Once we know the protonation state of each amino acid for the investigated conditions, we go on to perform all-atom MD simulations of a single mAb in explicit water and added salt to select characteristic molecular conformations to be used in many-protein MC simulations to study protein–protein interactions in more detail. Based on the information collected at both all-atom and amino-acid levels, we finally construct mesoscopic models made of simple beads which, while maintaining the Y-shaped geometrical anisotropy of the mAb, can be used to study the effect of coarse-graining on the resulting solution structure more efficiently. We, then, systematically vary the number of beads in the model to assess the optimal description of the molecules as a balance of accuracy and numerical cost. A schematic representation of the models used is presented in Fig. 1. The outcomes of these simulations are finally compared with SAXS data, as well as with the predictions from a simple and frequently used colloid model, to provide guidelines on the effects of coarse-graining on the resulting best-fit parameters used to define the potential of mean force between the mAbs.

## II. RESULTS AND DISCUSSION

### A. Calculation of amino acid charges

By using the one-protein MC simulations at the amino acid level, we estimate the mAb net charge, *Q_net_*, the mAb charge distributions, and the mAb electrostatic iso-surface at pH 6 and either 7 or 57 mM ionic strength. As described in Sec. IV, *Q_net_* is equilibrated using the titration moves. To start with, each amino acid has a negative charge. After a few steps of equilibration, the net charge quickly reaches a plateau, as reported in Fig. S1 of the supplementary material. The average values of the net charge of the mAb obtained discarding the first 200 equilibration steps are *Q_net_* = 31.3*e* for the *I* = 7 mM condition and *Q_net_* = 36.3*e* for *I* = 57 mM. This difference is expected due to salt screening effects which allow for a higher net-charge. In addition, we obtain the individual protonation state of the amino acid charge distribution that is then used as input in the all-atom MD simulations. In Figs. S2–S5, the charge iso-surface of the mAb is reported, illustrating the overall positive charge of the molecule. We remark on the different electrostatic properties of this antibody compared to previously studied mAbs,^14^ which results in a very different concentration behavior dominated by repulsive interactions. This feature is partially mitigated at high ionic strength where, despite an overall higher net charge, a larger degree of heterogeneity among the charges at the amino-acid level is estimated due to screening effects in different regions of the protein. This may also explain why self-assembly and an increased viscosity were observed experimentally at high ionic strength and very high mAb concentrations (see Ref. 8).

### B. mAb conformations obtained from MD simulations

**FIG. 2.**
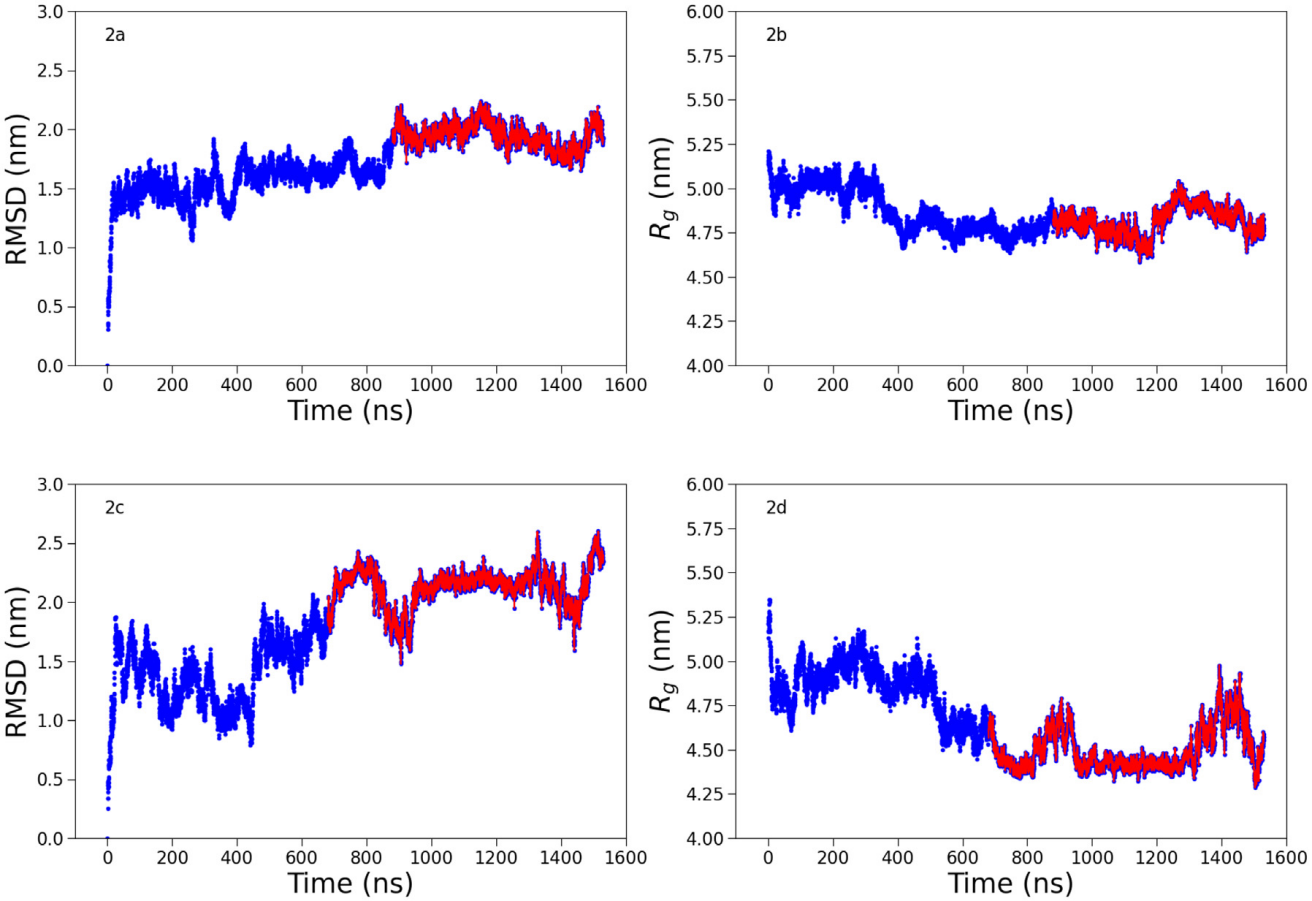
Root mean squared deviation (RMSD) and radius of gyration (*R_g_*) obtained from all-atom MD simulations for the condition I = 7 mM [(a) and (b)] and I = 57 mM [(c) and (d)]. The equilibrated part of the trajectory over which the average *R_g_* is calculated is highlighted in red.

**FIG. 3.**
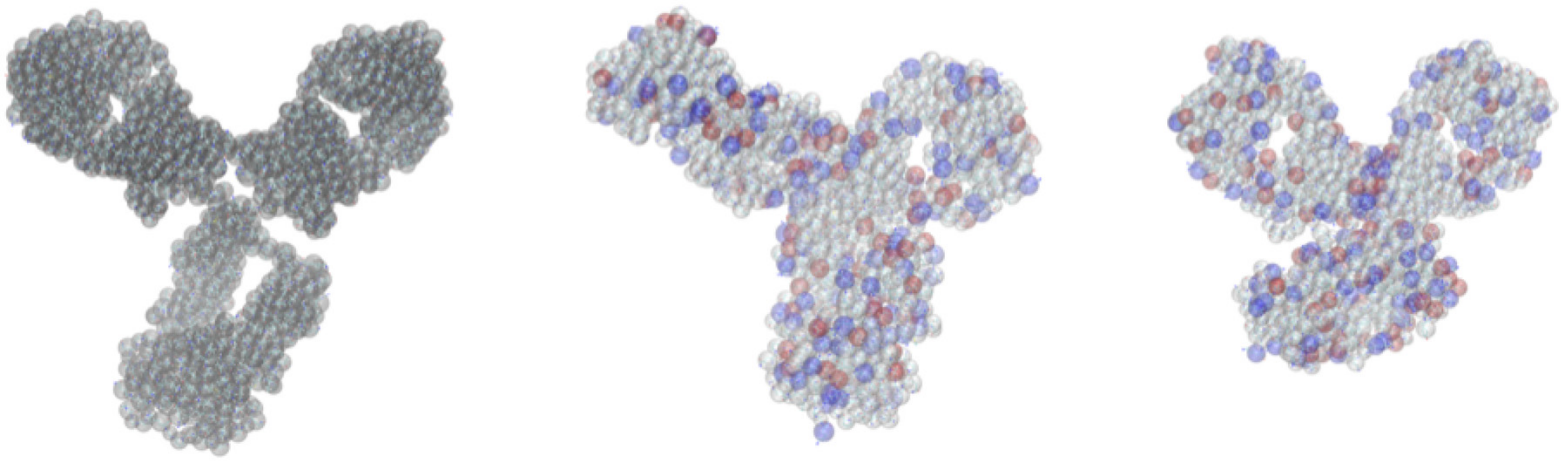
mAb structure before and after MD simulations. At the leftmost, the initial mAb structure at atomic resolution overlaps with its coarse-grained representation at the amino acid level. On the center and rightmost, the representative mAb conformations obtained from MD simulations, respectively, for the conditions at 7 and 57 mM of ionic strength. Red, blue, and gray beads represent negatively charged, positively charged, and neutral amino acids, respectively.

Next, we carry out all-atom MD simulations to obtain representative conformations of the mAb at both studied ionic strengths. In Fig. 2, we report the root-mean-squared deviation (RMSD) from the initial conformation, and the radius of gyration, *R_g_*, for the two investigated ionic strengths, *I*. For *I* = 7 mM, the RMSD converges approximately after 800 ns (part highlighted in red). Similarly, for *I* = 57 mM, the convergence starts approximately after 600 ns. Using the algorithm in Daura *et al.*^15^ the two MD trajectories are reduced into 11 and 20 groups of conformations, respectively, for the conditions *I* = 7 and *I* = 57 mM. The most probable conformations of these two groups are shown in Fig. 3 (center and rightmost) together with their representation at the amino acid level. We can see that in both cases, the average conformations are more compact compared to the initial structure obtained from homology modeling (see Fig. 3, leftmost). This is also manifested in their low *R_g_* value, which is found to be ∼4.8 nm for the condition at 7 mM and slightly lower, ∼4.5 nm, for the condition at 57 mM. It is interesting to compare the calculated form factors for the initial structure and the most probable conformations at low and high ionic strengths (both coarse-grained at the amino acid level) with experimental SAXS data obtained at low mAb concentrations (*C_p_* = 2 mg/mL), where structure factor effects should be minimal. The corresponding data are shown in Fig. S11 in the supplementary material and demonstrate that the agreement between the form factor from the relaxed structure and the experimental data is very good at low ionic strength except for very low q-values, where some non-negligible contributions from *S*(*q*) exist even at this low mAb concentration. Quite in contrast, the calculated form factor from the initial structure is in clear disagreement with the measured data, in particular, at *q*-values around 0.05−1 nm^−1^. For the higher ionic strength, the most probable conformation results in slightly less good agreement, in particular, at lower *q*-values, indicating that the simulated structure is slightly too compact, but the agreement with the experimental data is still much better than for the initial structure.

### C. Effective structure factor calculations from many-protein MC simulations at the amino-acid level

**FIG. 4.**
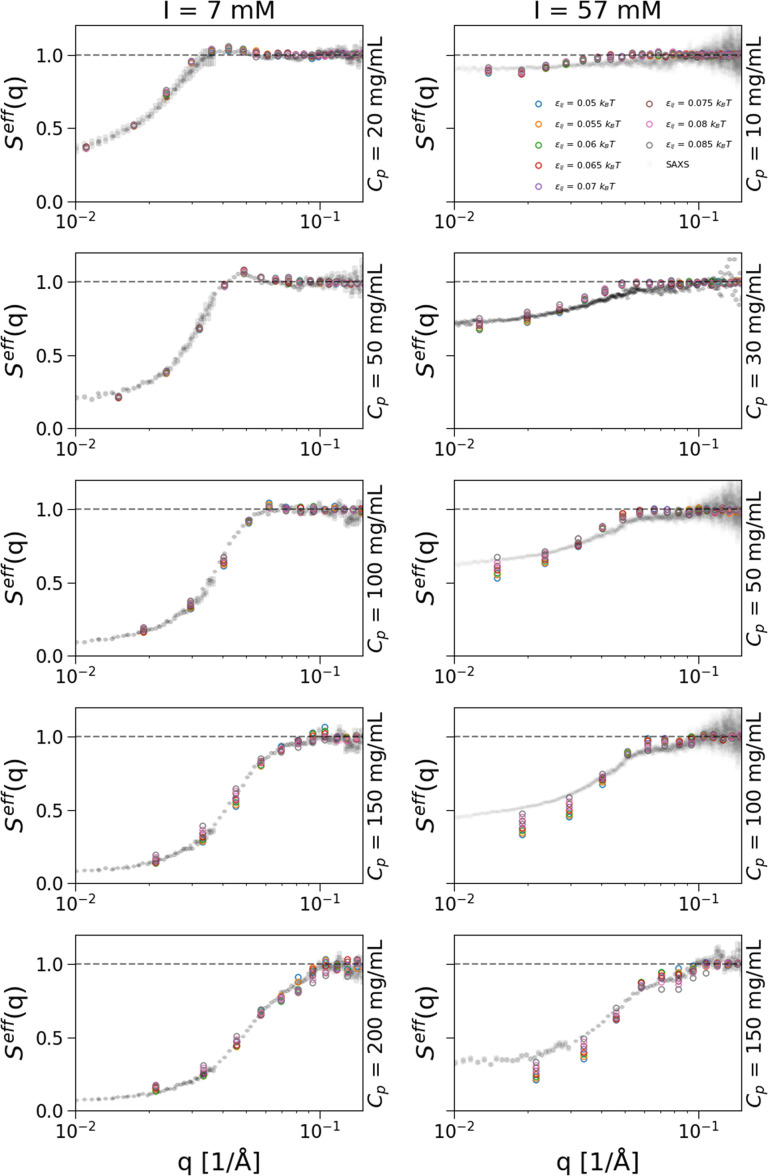
Effective structure factors, $S_{\text{eff}}(q)$, obtained from simulations of *N_p_* = 20 mAbs coarse-grained at an amino acid level in comparison with experimental data. The left and right columns refer to, respectively, the 7 and 57 mM ionic strength conditions, while on each graph the mAb concentration is indicated. The gray circles in the background represent the experimental data obtained by SAXS experiments together with their error bars, while the open symbols are the simulation results. Each of the colors indicates a different value of the depth of the attractive well in the Lennard–Jones term of Eq. (3), *ε_ij_*. The explored range is $\Delta \varepsilon_{ij}=$ 0.05–0.085 $k_{B}T$ with steps of 0.005 $k_{B}T$.

Using the two most representative conformations obtained from the MD simulations, we build a coarse-grained representation at an amino-acid level and perform many-protein MC simulations. We can, thus, calculate the $S_{\text{eff}}(q)$ of the mAb at different concentrations to be compared with experimental results. This is reported in Fig. 4 for the two series of simulations carried out at the two investigated ionic strengths. For each condition, we explored the effect of increasing the attractive well of Lennard–Jones potential, *ε_ij_*, from 0.05 $k_{B}T$, which is commonly used in coarse-grained simulations at the amino acid level as a starting value, to 0.085 $k_{B}T$. For the low ionic strength condition, left panels of Fig. 4, the simulation data obtained with the two most probable conformations show a very good quantitative agreement with the SAXS data at all studied mAb concentrations. We further note that the effect due to the increase in *ε_ij_* is almost negligible at low *C_p_* where all replicas, calculated for different values of *ε_ij_*, yield very similar results among each other. Hence, under these conditions, $S_{\text{eff}}(q)$ is completely dominated by long-range Coulomb interactions. This was expected, as by considering the electrostatic isosurface potential shown in Figs. S2 and S3 of the supplementary material it is clear that despite the mAb heterogeneous charge distribution the predominant influence is from the positive charges. Consequently, when other mAbs approach, they will encounter a relatively spherical +1 $k_{B}T/e$ repulsive isopotential surface, resulting in a quite globular centrosymmetric and soft repulsive interaction potential.

**FIG. 5.**
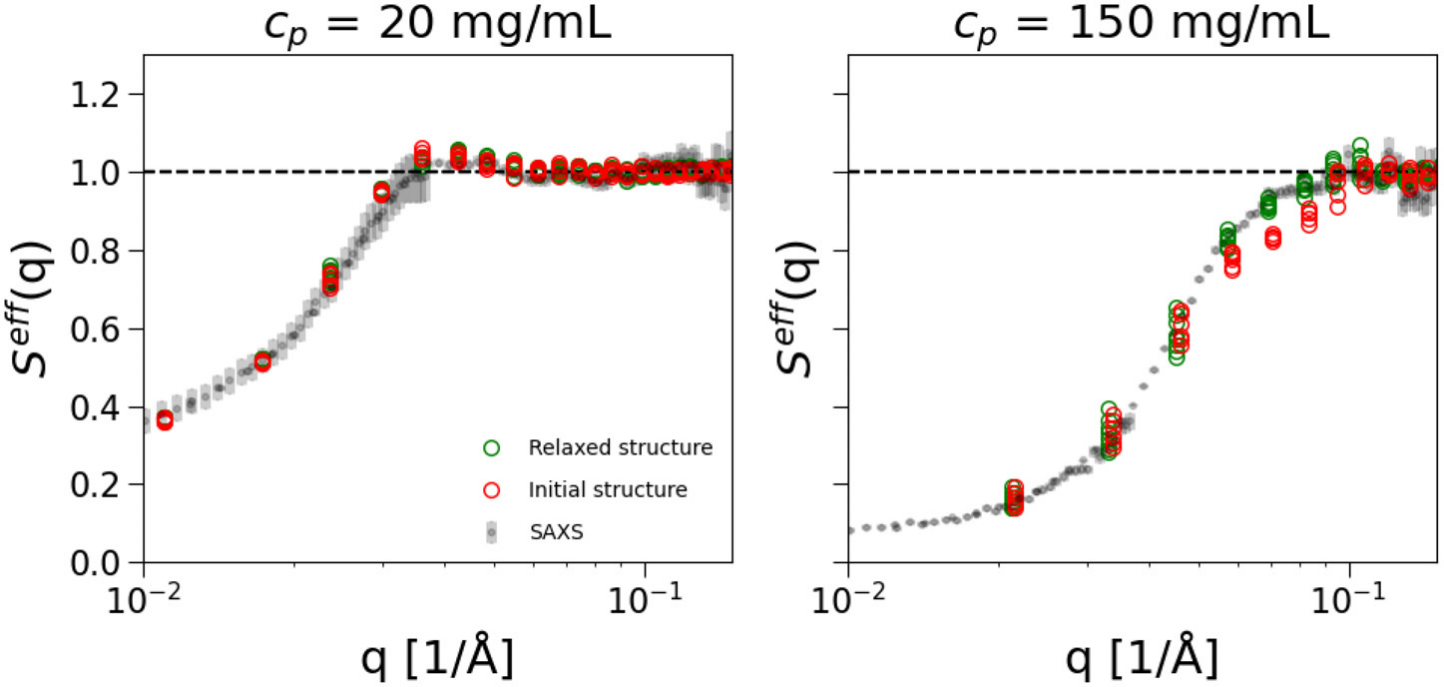
Effective structure factors, $S_{\text{eff}}(q)$, obtained from simulations of *N_p_* = 20 mAbs coarse-grained at an amino acid level and at low ionic strength (7 mM). Comparison with initial and relaxed structure. The explored range is $\Delta \varepsilon_{ij}=0.05-0.085k_{B}T$ with steps of 0.005 $k_{B}T$ for both cases.

At high mAb concentrations, an increase in *ε_ij_* has a visible effect as the different replicas give slightly different results. For the higher ionic strength (57 mM), right panels of Fig. 4, the first thing to notice is that the measured $S_{\text{eff}}(q)$ are overall less repulsive, compared to the low salt condition, as visible from the significant increase in the q→0 limit, that is directly related to the osmotic compressibility of the suspension. In addition, a small change in the attraction strength leads to a significant difference in the resulting $S_{\text{eff}}(q)$ even at the lowest studied mAb concentration (10 mg/mL). This effect increases with increasing *C_p_*. Under these concentrated conditions, the agreement between experiments and simulations is still good but it starts to deteriorate, especially in the low *q* limit. This could be due to the need to introduce flexibility in the model or to the absence of explicit counterions that could effectively change the screening conditions upon reversible interactions with the mAbs at this high ionic strength. In Fig. 5, we compare experimental results with simulation results obtained either using the initial non-relaxed PDB conformation or the relaxed conformation obtained by the all-atom MD simulations. The comparison is reported for two representative cases, respectively, at low (20 mg/mL on the left) and high mAb concentration (150 mg/mL on the right), both for the low ionic strength of 7 mM. For low *C_p_*, we find quantitative agreement with experimental data by using either the relaxed or the non-relaxed conformation. This result is likely because the mAb carries a high positive net charge and a relatively homogeneous charge distribution as demonstrated with the electrostatic potential iso-surfaces shown in Fig. S2 in the supplementary material. Therefore, electrostatic repulsions prevent contribution from short-range interactions where the actual mAb conformation plays a role. However, the situation is different at high mAb concentration, where, by using the initially not-relaxed conformation, $S_{\text{eff}}(q)$ is significantly underestimated in the *q*-range corresponding to the main (or nearest neighbor) peak, i.e., when mAbs are close to each other, suggesting that this conformation is overestimating the mAbs excluded volume. It is interesting to note that for these *q*-values around 0.05−1 nm^−1^ we also find significant discrepancies between the calculated form factor of the initial conformation and the measured SAXS data at low mAb concentrations (Fig. S11 in the supplementary material). These results put forward that an appropriate treatment of initial structures of the mAbs, although computationally demanding, is needed to correctly take into account the excluded volume, particularly at contact when attractive interactions play a role. This is confirmed by the fact that at high ionic strength, the comparison with experimental results further deteriorates in quality (not shown), which is the primary reason why we carried out all-atom MD simulations. On the other hand, a reassuring result is that, by using the most probable conformation, the coarse-grained many-body simulations are capable of reproducing the experimental results under the present conditions, except for the small discrepancy at high *I* and high *C_p_* discussed above.

### D. Effective structure factor calculation with bead models

By using a model at an amino acid level for the mAb representation, we have so far obtained a relatively accurate description of the interactions at play in the solution. However, due to the computational cost, we are limited in the number of mAbs we can simultaneously simulate, which is also directly related to the lowest *q*-value we can sample. To overcome this limitation, we further coarse-grain the mAb using the bead models shown in Fig. 1. For each of these models, we then perform many-protein MC simulations with different values for the effective charge, *Q_eff_*, and *ε_ij_*.

**FIG. 6.**
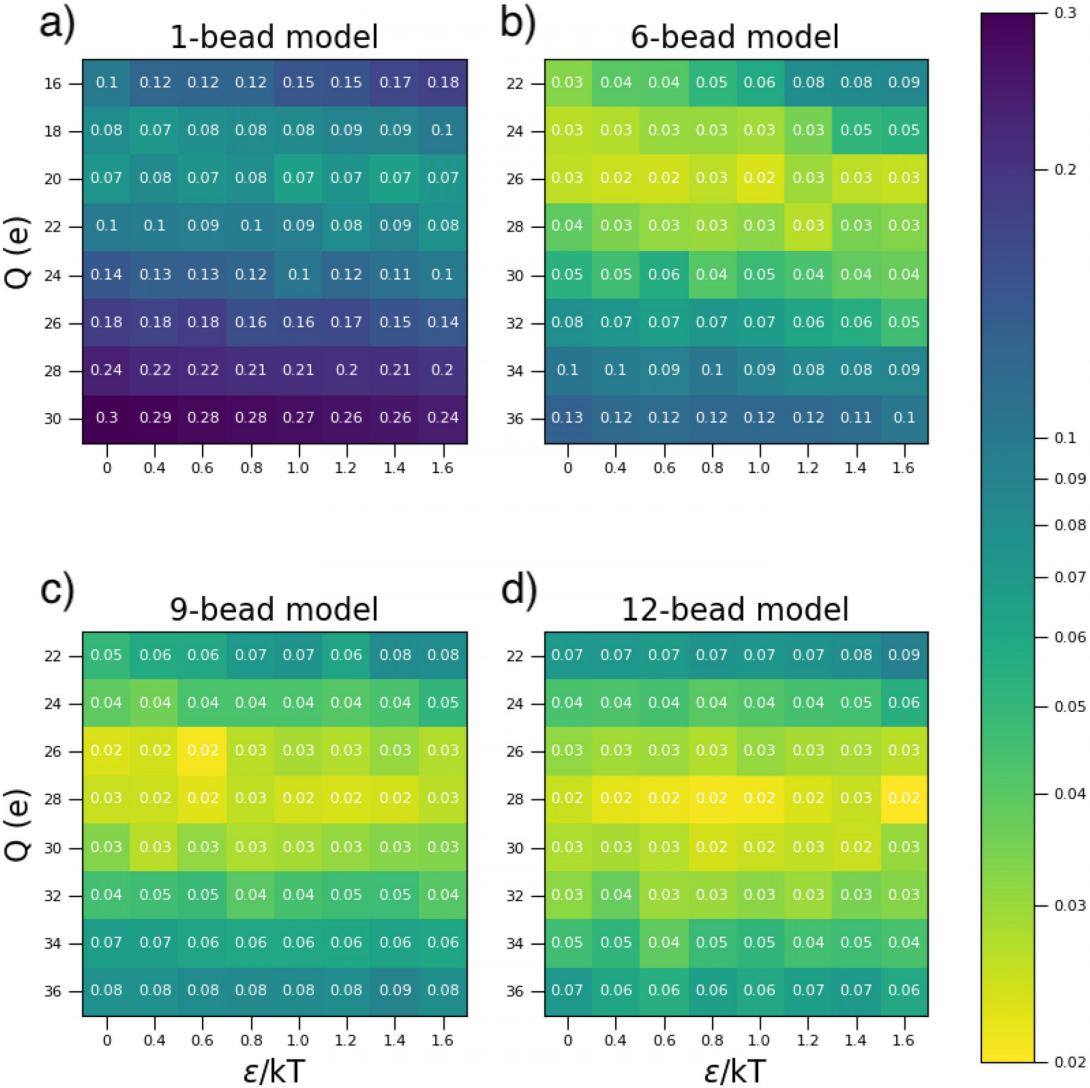
$\chi^{2}$ table for the 1-bead (a), 6-bead (b), 9-bead (c), and 12-bead (d) models related to the condition at low mAb concentration (20 mg/mL) and low ionic strength (7 mM) for several combinations of the potential parameters (*Q_eff_*, *ε_ij_*). Each $\chi^{2}$ value is associated with a color. A logarithmic scale is used to highlight differences among the values which are rounded to two decimal places.

In Fig. 6, we report for each of the studied models with different numbers of beads (1-bead, 6-bead, 9-bead, and 12-bead), the $\chi^{2}$ tables, measuring the deviation of the calculated $S_{\text{eff}}(q)$ from the experimental ones, to assess the ability of the chosen potential parameters (*Q_eff_*, *ε_ij_*) to reproduce the experimental data at low mAb concentration (20 mg/mL) and low ionic strength (7 mM). Each $\chi^{2}$ value is defined as

$$\chi^{2}=\sum_{i}\frac{{\left(S_{\text{eff}}^{\text{sim}}(q_{i})-S_{\text{eff}}^{\;\text{exp}\;}(q_{i})\right)}^{2}}{S{\left(q_{i}\right)}_{\text{eff}}^{\;\text{exp}}},$$ (1)

where $S{\left(q_{i}\right)}_{\text{eff}}^{\text{sim}}$ and $S{\left(q_{i}\right)}_{\text{eff}}^{\text{sim}}$ refer to the effective structure factor obtained, respectively, from simulations and SAXS experiments, and calculated in the point $q=q_{i}$. The number of *q* points goes from $q_{0}=0.01$ to $q_{50}=0.12$ Å^−1^ with a Δq between each point equal to 0.0022 Å^−1^.

**FIG. 7.**
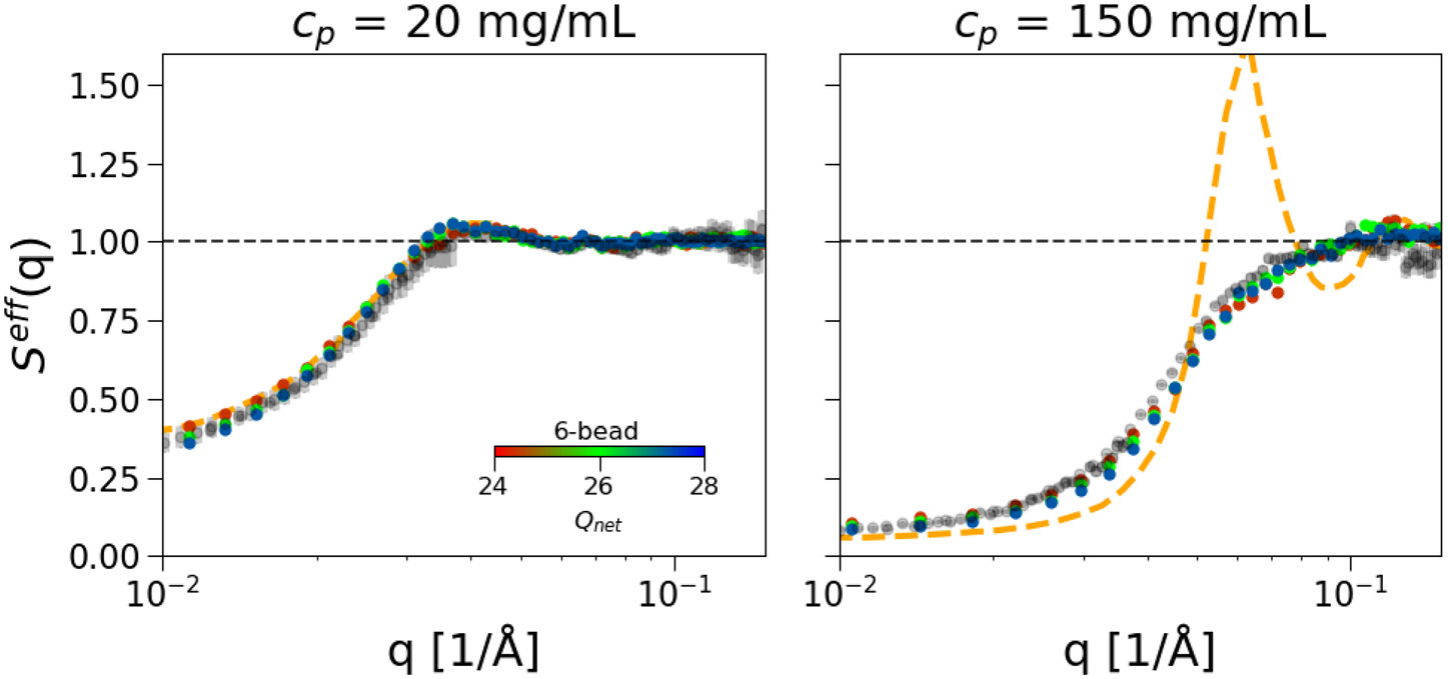
Effective structure factors, $S_{\text{eff}}(q)$, obtained from experimental data (gray circles) at low ionic strength (7 mM) and 20 mg/mL (left) and 150 mg/mL (right) mAb concentrations in comparison with the 1-bead model (dashed orange line) and an effective charge of *Q_eff_* = 20 *e* and the 6-bead model (open circles) and an effective charge in the range $\Delta Q_{\text{eff}}=26\pm 2$*e.*

We also report the tables for all the other studied conditions in the supplementary material for completeness (Figs. S6–S9). These tables provide us with a graphical indication of the uniqueness of a given combination of *Q_eff_* and *ε_ij_* to best reproduce the experimental data, where the lower the $\chi^{2}$ value, the better that particular combination of charge and attractive strength reproduces the experimental data. Figure 7 clearly shows that while *Q_eff_* is well defined under these conditions of low *C_p_* and ionic strength, *ε_ij_* has no systematic effect and can, thus, not be determined accurately for these solutions. Moreover, *Q_eff_* strongly depends on the model. For the 1-bead model, the value of *Q_eff_* best reproducing the experimental results is found to be in the range $\Delta Q_{\text{net}}=20\pm 1.5$*e* (see Fig. 6), while for the 6-bead model, this value shifts to 26 ± 2*e* (see Fig. 7).

The $S_{\text{eff}}(q)$ obtained with these effective charges in combination with $\varepsilon_{ij}=0.8k_{B}T$ are reported in Fig. 7 for the conditions at low ionic strength (7 mM) and both low (20 mg/mL) and high (150 mg/mL) mAb concentrations. At low mAb concentration (Fig. 7, left), both the 1-bead and the six-bead models are in quantitative agreement with the experimental data. However, the net charge we have to assign to match the experimental data is significantly smaller than that expected from the model at the amino acid level and our constant-pH Monte Carlo simulations. At high mAb concentration (Fig. 7, right), instead the 1-bead model is unable to reproduce the experimental behavior. Indeed, $S_{\text{eff}}(q)$ takes the shape that is typical for hard-sphere charged colloids at low ionic strength, with a highly enhanced first peak. Differently, the 6-bead model yields quite good agreement with experimental data, except for the q-range 0.03 ≲ q ≲ 0.07 Å^−1^, where $S_{\text{eff}}(q)$ is slightly underestimated. Now increasing the number of beads, the $\chi^{2}$ tables in Fig. 6 suggest that $\Delta Q_{\text{net}}=28\pm 2$*e* and $\Delta Q_{\text{net}}=29\pm 2$*e*, respectively, for the 9-bead and the 12-bead models. These results indicate a clear trend: as the number of beads increases, the window of effective charges able to reproduce the experimental data increases, approaching the expected value estimated at the amino acid level. This is illustrated in Fig. 9, which also shows that for a number of beads greater than 9, *Q_eff_* saturates, suggesting no major differences between the two models. This is confirmed by Fig. S10 (in the supplementary material) where the comparison of $S_{\text{eff}}(q)$ between the 9-bead and the 12-bead models is reported for some of the studied conditions, showing indistinguishable results for the two bead models. Hence, we can conclude that the 9-bead is the best compromise in balancing computational cost and accuracy. It is also interesting to note that the agreement between simulated and measured $S_{\text{eff}}(q)$ at high mAb concentrations is significantly better for the much more strongly coarse-grained 6 or 9-bead models than for the amino acid-level coarse-grained model when using the initial non-relaxed structure (see Figs. 5, 7, and 10. While this seems at first counterintuitive, at high mAb concentrations excluded volume interactions and the actual dimensions of the mAb structure become important. A direct comparison between the initial and the relaxed configuration at the full amino-acid level and the 6-bead model as shown in Fig. S12 (in the supplementary material) indeed indicates that the agreement between the 6-bead model and the relaxed structure is improved when compared with the initial non-relaxed structure. As a result, the simpler Y-shaped bead models perform better than the initial configuration in terms of the local structural correlations at very high mAb concentrations. It appears that the appropriate choice of the radius of gyration is crucial to correctly reproduce the overall dimensions and local packing constraints when using highly coarse-grained bead models. Having focused on the low ionic strength, we now turn to the I = 57 mM case, where repulsion is much more screened, and also the amino acid model showed some discrepancy from the experimental results at high mAb concentrations. It is important to note that to tackle this case, we should, in principle, allow for a different net charge since this is also found to be different in the constant-pH simulations. We then need to adjust the Debye length according to Eq. (3) to take into account the added NaCl as well as the counterions from the charged beads. On the other hand, we expect the attractive strength *ε_ij_* to only weakly depend on the ionic strength. We, thus, fix it to the same value that provided the best agreement for I = 7 mM, namely, $\varepsilon_{ij}=0.8$ k_B_T. In Fig. 10, we report $S_{\text{eff}}(q)$ for the 9-bead model in comparison with the SAXS data for both ionic strength conditions (7 and 57 mM) and different mAb concentrations. We compare results for different *Q_eff_*, finding no major differences between the two cases. Overall, we find that the agreement between experimental and numerical structure factors is quantitative at all studied conditions with small discrepancies observed at high mAb concentration and high ionic strength. This is probably because, under these conditions of high screening, attractive interactions become more important and these may be anisotropic and/or localized in the more hydrophobic spots of the mAb, something we cannot properly capture by assigning an overall identical charge to all beads and an isotropic long-range attraction. However, the quality of the agreement is comparable to that obtained for the amino acid level description (see Fig. 4).

**FIG. 8.**
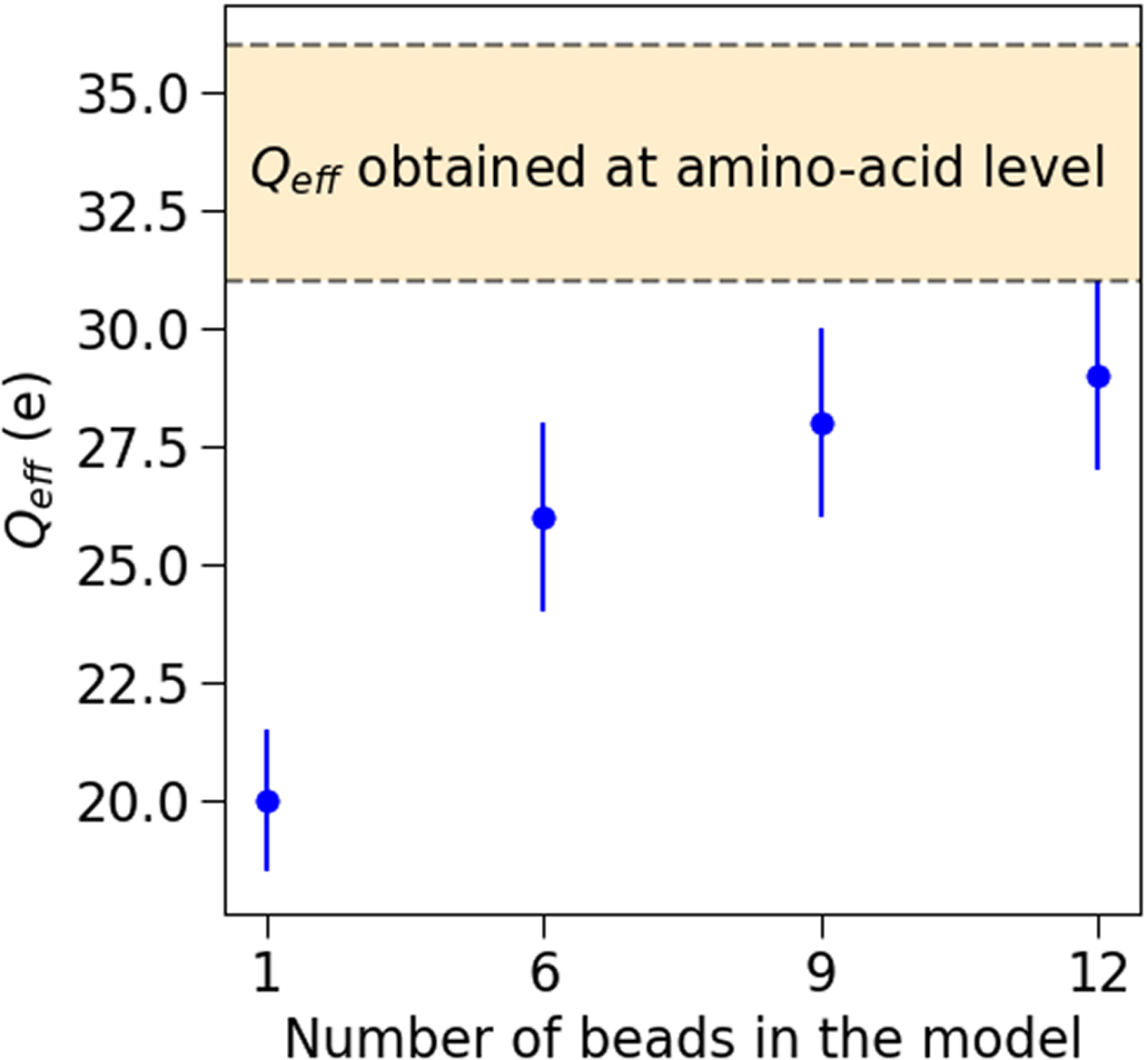
Values of *Q_eff_* best reproducing the experimental data for the different bead models used as judged from the -tables shown in Figs. 6 and S6–S9 in the supplementary material.

**FIG. 9.**
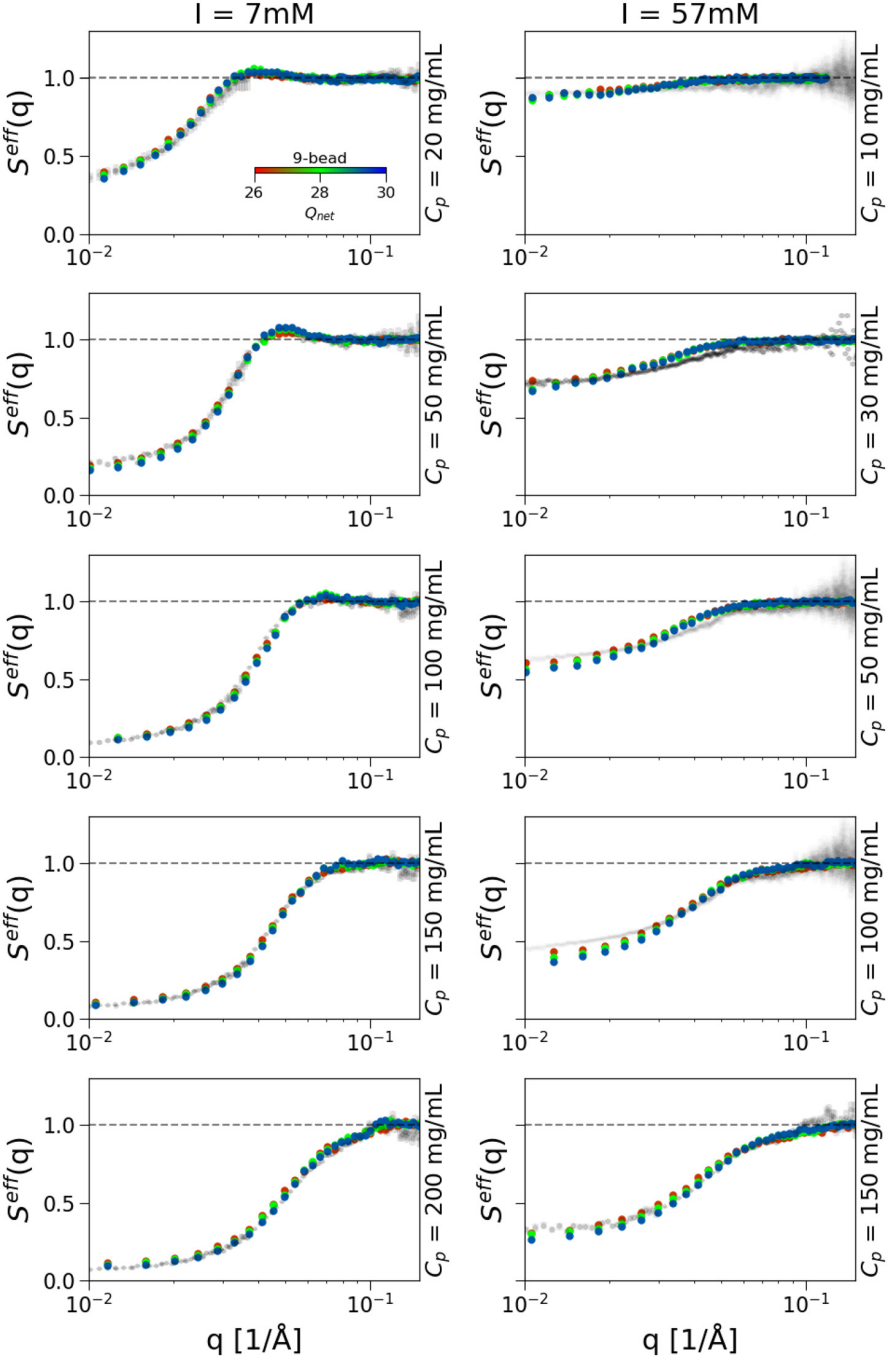
Effective structure factors $S_{\text{eff}}(q)$, obtained from simulations with the 9-bead model and an effective charge in the range of $\Delta Q_{\text{eff}}=26-30$*e* in comparison with experimental data (gray circles). Here, the attractive term is van der Waals-like with $\epsilon_{ij}=0.8$*k_B_T.*

## III. CONCLUSIONS

In this study, we aimed to develop a computationally efficient protocol for investigating protein solutions, specifically monoclonal antibodies (mAbs), and reproduce their structural properties accurately. We exploited a multi-scale approach, spanning from all-atom MD simulations and coarse-graining at amino acid level, to build up mesoscale bead models which turned out to be very efficient in reducing the system complexity while preserving the essential structural characteristics. Our investigations focused on a mostly repulsive mAb with an isotropic charge distribution and a high net charge. By using a coarse-graining at the amino acid level in combination with constant-pH MC simulations, we calculated the mAb charge distribution for the selected solution conditions. Then, we used the knowledge acquired to perform all-atom MD simulations of a single mAb and extrapolate representative mAb conformations. This step turned out to be necessary since we have shown that performing many-protein simulations at high concentrations with a non-relaxed mAb conformation leads to an incorrect estimate of the excluded volume. With such representative conformations coarse-grained at an amino acid level, we then carried out many protein MC simulations to reproduce a wide range of experimental structure factors up to the mAb concentration of 200 mg/mL. However, simulating mAb solutions at this level of detail is computationally demanding, especially for studying behavior in the high-concentration regime. Therefore, based on the information extrapolated at both all-atom and amino-acid levels, we have built up bead models much easier to handle and at the same time very accurate in describing the structural properties of the mAb solution.

The bead models rely on two key parameters: the effective charge *Q_eff_*, which is isotropically distributed among the beads, and the strength of attraction *ε_ij_*, which takes into account short-range interactions on top of the excluded volume, that is taken care of by the number of beads in the model as explained above. To select *Q_eff_* for each bead model, we focused on low mAb concentration and low ionic strength, where electrostatic repulsions are dominant. By minimizing the difference between experimental data and model predictions, we determined the appropriate *Q_eff_* value, which is also found to approach the value obtained in the one-body constant-pH simulations with an increasing number of beads. For *ε_ij_*, we then found the best value that, in combination with *Q_eff_*, can reproduce the experimental data under all studied conditions of mAb concentration and ionic strength. It is, however, important to note that in contrast to *Q_eff_*, *ε_ij_* cannot accurately be determined independently from a measurement of $S_{\text{eff}}(q)$ as these parameters are strongly coupled as demonstrated by the $\chi^{2}$-tables shown in the supplementary material.

By systematically increasing the level of detail, we have identified the minimum bead model capable of fully reproducing solution structure factors for mAb concentrations up to 200 mg/mL and ionic strengths up to 57 mM. In more detail, the 1-bead model was able to reproduce the experimental structure factor only at low mAb concentration and low ionic strength. This is because, in this regime, the interactions are mainly driven by strong long-range electrostatic repulsions such that short-range interactions and the actual shape of the molecule only play a minor role. On the other hand, the Y-shaped 6-bead model provided a significant improvement, confirming the need to include shape anisotropy to account for local structural correlations at mAb concentrations exceeding 50 mg/mL. However, such a model failed to reproduce the effective structure factor at mAb concentrations above 150 mg/mL, due to overestimation of excluded volume effects. Both the 9-bead and 12-bead models were shown to be able to resolve this problem with almost identical accuracy, so we can conclude that the 9-bead is the best compromise in terms of gain of computational cost, in agreement with recent work on a different, more attractive and cluster-forming mAB.^14^

The validation of the coarse-grained bead models was carried out against small-angle x-ray scattering (SAXS) experiments and the comparison with amino-acid level descriptions highlighted the computational advantage of the former models. In conclusion, our developed bead models offer a computationally affordable yet sufficiently accurate approach to studying mAb solutions' structural properties. Their efficiency allows for extended investigations under various conditions, facilitating predictions of structure factors for challenging concentration regimes not easily accessible in experimental setups. Future work will need to extend and generalize such simplified treatments to more complicated mAbs where aggregation and/or self-assembly also take place, taking into account electrostatics in an accurate way to include heterogeneous charge distributions and to allow for, e.g., directional attraction, which often drives cluster formation in mAbs.^14,16^

## IV. METHODS

### A. Initial structure calculation

We use the IGg1 mAb called Actemra (or Tocilizumab) as the basis of our work, for which we have described a detailed characterization of key solution parameters over a large range of concentrations and solution conditions elsewhere.^8^ The primary amino acid sequence was retrieved from patent US20120301460. A homology model was prepared using the Antibody Modeler module in Molecular Operating Environment (MOE) 2020.^8,13^ Briefly, the primary sequence was used to identify suitable existing structures for the framework and variable domains upon which the model was built. The complementarity-determining regions (CDRs) were modeled individually based on known loop structures and were then grafted onto the antibody framework. The structure then underwent energy minimization using “LowModeMD” to eliminate steric clashes.

### B. One-protein MC simulations at the amino acid level

Based on this structure, and using the same protocol as in Ref. 14, we construct a coarse-grained representation of the mAb by replacing each amino acid with a spherical bead of diameter $\sigma_{\text{bead}}^{aa}={\left(6M_{W}/\pi \rho \right)}^{1/3}$, where *M_W_* is the amino acid molecular weight (in g mol^−1^), *ρ* = 1 (in g mol^−1 ^Å^−3^) is an average amino acid density,^17^ and the suffix *aa* stands for “amino acid” (see Fig. 3, leftmost).

With the amino-acid-based coarse-grained model, we perform Metropolis-Hastings Monte Carlo (MC) simulations of the mAb solution using Faunus (v2.9.1 git 3edf85cf),^18^ which is a software allowing for several types of MC simulations, to estimate the mAb charge distribution (as performed here^19^). In particular, we carry out constant-pH MC simulations with a single rigid mAb and titration moves only,^20^ which allow the amino-acid charges to fluctuate and to reach an equilibrium distribution at a given pH and the ionic strength. We consider two ionic strength conditions as in experiments:^8^ 7 and 57 mM. For each of them, we perform a simulation of 10^4^ MC sweeps where, on each sweep, *N* = 10 titration moves were attempted. The full interaction potential is defined as

$$\begin{array}{c} \beta V=\underset{\mathrm{Electrostatic}}{\underset{︸}{\sum_{i}^{n-1}\sum_{j=i+1}^{n}\frac{\lambda_{B}q_{i}q_{j}}{r_{ij}}e^{-r_{ij}/\lambda_{D}}}}\\ \underset{\mathrm{Titration}}{\underset{︸}{-\sum_{i}\;\text{ln}\;\left(\frac{N_{i}!}{(N_{i}+\nu )!}V^{\nu_{i}}\right)-\sum_{i}\;\text{ln}\;(10)(pK_{a,i}-pH)}}, \end{array}$$ (2)

where the first term accounts for electrostatic interactions; the second and the third terms account for the energy due to the titration of charged amino acids. Here, $\beta =1/k_{B}T$ is the reciprocal of the thermal energy, where *k_B_* is the Boltzmann constant and *T* is the system temperature; $\lambda_{B}=\beta e^{2}/4\pi \varepsilon_{0}\varepsilon_{r}=7.1$ Å is the Bjerrum length, where *e* is the electron charge, *ε*_0_ is the vacuum permittivity, and $\varepsilon_{r}=78.7$ is the relative dielectric constant of water at room temperature ( 25 °C) (as the solvent is treated as a continuum); *q_i_* and *q_j_* are the electrical charges on the *i*th and *j*th amino acid, and *r_ij_* is the distance between them; and *λ_D_* is the Debye length, which accounts for charge screening effects as the salt is treated implicitly. As we consider single molecule behavior only, i.e., work in the limit of infinite dilution, *λ_D_* is set equal to $3.04/\sqrt{I(M)}$ Å,^21^ where *I*(*M*) is the molar ionic strength. The titration terms propagate the reaction AH⇄A+H back and forward using a reactive MC scheme.^20^ Here, *AH* is the protonated form of the amino acid, while *A* + *H* is its dissociated form. In addition, *N_i_* is the number of charged amino acids of species i, *ν_i_* is the stoichiometric coefficient (positive for the products and negative for the reagents), *V* is the volume of the system, and $K_{a,i}$ is the acid dissociation constant of the *i*th amino acid residue. In this way, the MC moves shift the equilibrium reaction based on the solution conditions (such as pH and salt concentration). The $pK_{a,i}$ values are taken from the literature^22^ and reported in Table S1 of the supplementary material.

### C. All-atom molecular dynamics simulations and conformational analysis

Next, we perform two molecular dynamics (MD) simulations of a single mAb, one for each ionic strength condition (7 mM or 57 mM), with the GROMACS package (version 2022.2),^23^ using the Amber99SB force field^24^ and the SPC/E water model.^25^ The protonation state of charged amino acids, histidine, arginine, lysine, aspartic acid, glutamic acid, and glutamine, have been assigned based on the average charges obtained for each amino acid from the one-protein MC simulations at pH 6 and either 7 mM or 57 mM ionic strengths.

The mAb is inserted into a cubic simulation box of volume 17.85 × 17.85 × 17.85 nm^3^, containing either 183 792 or 183 436 water molecules, based on the ionic strength condition. In each simulation, the net charge is neutralized by adding an appropriate number of counterions. Furthermore, we add the necessary amount of Na^+^ and Cl^−^ ions to reach the desired ionic strength. Before each production run, the system energy of the whole system is minimized using the steepest descent method with a tolerance of 1000 kJ mol^−1 ^nm^−1^. We equilibrate the temperature and pressure of the system in two phases. First, using the Berendsen thermostat,^26^ we stabilize the system temperature at 300 K performing a run of 100 ps in the canonical ensemble. Then, we equilibrate the pressure with the Parrinello–Rahman method^27^ to the target value of 1 bar. After the equilibration process, we carry out simulations at each condition using the leap-frog integrator^28^ for a total time of 1.5 *μ*s. All simulations are performed with an integration step of 2 fs and with a 1 nm cutoff for the short-range van der Waals (vdW) interactions, while electrostatics are treated using the particle mesh Ewald^29^ (PME) method with a real space cutoff of 1 nm. Periodic boundary conditions (PBCs) are used. To satisfy the so-called minimum image convention, i.e., to avoid the mAb seeing its periodic image, which would lead to spurious forces, we specify a solute-box distance of 1.0 nm. This ensures at least 2.0 nm between any two periodic images of the mAb.

By analyzing the MD trajectories, we selected representative conformations of the mAb using the algorithm described by Daura *et al.*^15^ This is a well-established method to analyze MD trajectories and to identify distinct conformational states of a bio-molecule. It allows the grouping of similar protein configurations based on their structural similarity. The algorithm is mainly based on three different steps: (i) measuring the structural differences between all conformation pairs by calculating the root mean squared deviation (RMSD); (ii) constructing a similarity matrix where it assigns higher similarity values to pairs of conformation with lower RMSD values. In this process, a cutoff is selected to determine the maximum distance at which two conformations are considered to be similar. Here, we use a cutoff of 6 nm; and (iii) grouping similar conformations, i.e., selecting those with relative distance in the RMSD smaller than the cutoff, to find distinct structural states. For each simulation (one for each ionic strength), the MD trajectory is then reduced to groups of conformations. Each group has a central structure that is the closest (the most similar) to all the others in the same group (in terms of RMSD). We chose the central structure of the most populated group of each simulation as the representative for the entire MD trajectory.

### D. Many-protein MC simulations at the amino-acid level

The most representative conformation obtained from the analysis of the all-atom MD trajectories was coarse-grained at an amino-acid level for each of the two ionic strengths investigated (see Fig. 3, center and rightmost) and used to perform many-protein MC simulations. In more detail, *N_p_* = 20 rigid translating and rotating mAbs are inserted in a cubic box of volume $V=N_{p}M_{w}/(c_{p}N_{a}1\times 10^{-27})$, where V is in Å^3^, *M_w_* = 148 kDa (or, equivalently, 148 000 g/mol) is the mAb molecular weight, *N_a_* is Avogadro's number, and *C_p_* is the mAb concentration that we chose equal to 20, 50, 100, 150, and 200 mg/mL to compare with experimental data.^8^ The interactions are ruled by the pair potential between the *i*th and *j*th amino acids, $V(r_{ij})$, defined as

$$\beta V(r_{ij})=\underset{\mathrm{Electrostatic}}{\underset{︸}{\lambda_{B}q_{i}q_{j}\frac{e^{-r_{ij}/\lambda_{D}}}{r_{ij}}}}+\underset{\mathrm{vdW}}{\underset{︸}{4\epsilon_{ij}\left[{\left(\frac{\sigma_{ij}}{r_{ij}}\right)}^{12}-{\left(\frac{\sigma_{ij}}{r_{ij}}\right)}^{6}\right]}}.$$ (3)

The amino acid potential is composed of the sum of two accounting terms for electrostatic and van der Waals interactions. For a solution containing charged mAbs, dissociated counterions, monovalent salt, and buffer ions, $1/\lambda_{D}^{2}$ is calculated as

$$1/\lambda_{D}^{2}=4\pi \lambda_{B}\left[\left(\frac{1}{1-\phi }\right)Z_{\mathrm{mAb}}\rho_{\mathrm{mAb}}+2\rho_{\text{salt}}+2\rho_{\text{buffer}}\right],$$ (4)

where ϕ is the excluded volume of a single mAb and the factor 1/(1−ϕ) accounts for the free accessible volume, with ϕ being calculated as the excluded volume of a hard sphere of diameter *σ_HS_*, equal to

$$\phi_{HS}=\rho_{\mathrm{mAb}}\frac{\pi \sigma_{HS}^{3}}{6},$$ (5)

where *ρ_mAb_* is the mAb number density. For our simulations, we choose *σ_HS_* = $2R_{g}$, where *R_g_* is the average radius of gyration of the mAb obtained from MD simulations. For the two ionic strength conditions considered, *R_g_* is equal to 48.2 Å for *I* = 7 mM and 45 Å for *I* = 57 mM.

Furthermore, *ρ_salt_* and *ρ_buffer_* are the number densities of salt and of the dissociated buffer ions, respectively. The van der Waals term accounts for short-range interactions, which is here represented by the Lennard–Jones potential, where *ε_ij_* is the depth of the attractive well, and $\sigma_{ij}=(\sigma_{\text{bead},i}^{aa}+\sigma_{\text{bead},j}^{aa})/2$ is the characteristic interaction distance.^30^ For each of the solution conditions, we perform eight replicas with the replica exchange method,^31^ which allows us to reach a faster sampling, especially for high mAb concentration conditions. Each of the replicas is characterized by the assigned value of *ε_ij_*. By increasing *ε_ij_* among the replicas by 0.005 $k_{B}T$, we explore the range $\Delta \varepsilon_{ij}=$ 0.05–0.085 $k_{B}T$. This interval has been selected from previous studies of coarse-grained models for both globular proteins^32–36^ and antibody solutions.^12,16,19^ For simplicity, at this stage, we neglect the hydrophilic and/or hydrophobic nature of the single amino acid and we assume that each bead contributes with the same attractive strength. Each simulation is carried out for 10^4^ MC sweeps where, on each sweep, each protein is attempted to be translated and rotated.

### E. Many-protein MC simulations with bead-models

In parallel, we also develop a different strategy to investigate the mAb solutions, which consists of simplifying the mAb representation with simple models made by a limited number of beads. Specifically, we use models with one, six, nine, and twelve beads, each constituted by either one or several identical hard spheres with an effective diameter, *σ_bead_* (see Fig. 1). Except for the 1-bead model, we assume a fixed angle of 60° between the two arms of the Y-shaped mAb (the FAB domains) and we choose *σ_bead_* to reproduce the same average radius of gyration, *R_g_*, obtained from all-atom MD simulations. Since *R_g_* is slightly different based on the two ionic strength conditions, for each of the bead models, we select two *σ_bead_* values, which are reported in Table S2 of the supplementary material.

The bead–bead pair potential, $V(r_{ij})$ is the sum of a hard-sphere contribution plus electrostatic and van der Waals interactions taking place at bead–bead distances, *r_ij_*, longer than *σ_bead_*, as

$$\begin{array}{c} \beta V(r_{ij})=\infty \;\;\;\;\;\;\;\;\;\;\;\;\;\;\;\;\;\;\;\;\;\;\;\;\;\;\;\;(r_{ij}<\sigma_{\text{bead}})\\ =\underset{\mathrm{Electrostatic}}{\underset{︸}{\lambda_{B}q_{\text{bead}}^{2}{\left(\frac{e^{\sigma_{\text{bead}/2\lambda_{D}}}}{1+\sigma_{\text{bead}}/2\lambda_{D}}\right)}^{2}\frac{e^{-r_{ij}/\lambda_{D}}}{r_{ij}}}}-\underset{\mathrm{vdW}}{\underset{︸}{\epsilon_{ij}{\left(\frac{\sigma_{\text{bead}}}{r_{ij}}\right)}^{6}}}(r_{ij}>\sigma_{\text{bead}}), \end{array}$$ (6)

where, in the electrostatic term, *q_bead_* = *Q_eff_*/*N_beads_* is the electrical charge carried by each bead, with *N_beads_* being the number of beads present on each model, and *Q_eff_* being the net effective charge of the mAb. The inverse of the Debye screening length, $1/\lambda_{D}$, is again given by Eq. (4). Since the beads are treated as hard spheres, only the attractive part of the Lennard–Jones potential is included to account for short-range interactions. As for the simulations at the amino-acid level, for each condition, we use a cubic simulation box of volume $V=N_{p}M_{w}/(c_{p}N_{a})$, where now *N_p_* is set to 500. Each simulation is carried out for 10^5^ MC sweeps, where on each sweep, each molecule is attempted to be rotated and translated.

### F. Effective structure factor calculation

The purpose of all our many-body simulations is to obtain the effective structure factor, $S_{\text{eff}}(q)$ of the mAb solution under different conditions. $S_{\text{eff}}(q)$ is calculated explicitly as^31,37^

$$S_{\text{eff}}(q)=\frac{1}{\left\langle F(q)\right\rangle }\frac{1}{N_{\text{tot}}}\left\langle {\left(\sum_{i}^{N_{\text{tot}}}\;\text{sin}\;(qr_{i})\right)}^{2}+{\left(\sum_{i}^{N_{\text{tot}}}\;\text{cos}\;(qr_{j})\right)}^{2}\right\rangle ,$$ (7)

where *N_tot_* is the product of the number of mAbs and the number of beads used in each model (i.e., 1, 6, 9, …); *r_i_* and *r_j_* identify the position of the *i*th and *j*th bead, and *F*(*q*) is the form factor that is obtained from an MC simulation in a cubic box of volume *V* where only a single molecule is inserted (*N_p_* = 1). The averages are performed over all directions obtained by permuting the crystallographic index [100], [110], and [111] of the cubic box to define the scattering vector q=2πp/L(h,k,l), where $p=1,2,\ldots ,p_{\text{max}}$, and *p_max_* = 25.

## SUPPLEMENTARY MATERIAL

See the supplementary material for the following additional information: A table with all the pK values of the different amino acids used in the Metropolis–Hastings Monte Carlo (MC) simulations performed to determine the charge distribution on the mAb for both ionic strengths (Table S1); an example of the net charge equilibration for both ionic strengths (Fig. S1); examples of the electrostatic iso-potential surfaces for ±1 and 3 $k_{B}/e$ at 7 and 57 mM ionic strengths (Figs. S2–S5); the $\chi^{2}$-tables for all bead models and mAb concentrations investigated (Figs. S6–S9); a comparison of the effective structure factor $S_{\text{eff}}(q)$ obtained from simulations of the 9 and 12-bead models for two different mAb concentrations at both ionic strength values (Fig. S10); a comparison between the experimental SAXS data and the calculated form factor for the initial structure obtained from homology modeling and the relaxed structure from the MD simulations at 7 and 57 mM ionic strength (Fig. S11); and a comparison between the coarse grained (at the amino acid level) structure for the initial and the relaxed structure at 7 mM ionic strength and the 6-bead model (Fig. S12).

## Data Availability

The data that support the findings of this study are openly available in Zenodo at https://doi.org/10.5281/zenodo.10478576, Ref. 38.
